# Polygenic score from MODY genes is associated with type 1 diabetes and disease characteristics

**DOI:** 10.1007/s00592-025-02544-w

**Published:** 2025-06-20

**Authors:** Eulalia Catamo, Andrea Conti, Roberto Franceschi, Klemen Dovc, Camilla Morosini, Davide Tinti, Luana Aldegheri, Stefania Cappellani, Gianluca Tamaro, Angela Zanfardino, Elena Faleschini, Ivana Rabbone, Riccardo Bonfanti, Tadej Battelino, Dario Iafusco, Gianluca Tornese, Antonietta Robino

**Affiliations:** 1 Institute for Maternal and Child Health – IRCCS Burlo Garofolo, Via Dell’Istria 65, Trieste, Italy; 2 Division of Pediatrics, S. Chiara General Hospital, APSS, Trento, Italy; 3 Faculty of Medicine, University of Ljubljana, Ljubljana, Slovenia; 4 Department of Endocrinology, Diabetes and Metabolism, University Children’s Hospital, University Medical Centre Ljubljana, Ljubljana, Slovenia; 5 Diabetes Research Institute, Department of Pediatrics, IRCCS San Raffaele Hospital, Milan, Italy; 6 Center for Pediatric Diabetology, A.O.U. Città Della Salute e Della Scienza, Turin, Italy; 7 Department of Woman, Child and of General and Specialized Surgery, Università Degli Studi Della Campania “Luigi Vanvitelli”, Naples, Italy; 8 Division of Pediatrics, Department of Health Sciences, University of Eastern Piedmont, Novara, Italy; 9 Department of Medicine, Surgery and Health Sciences, University of Trieste, Trieste, Italy

**Keywords:** Type 1 diabetes, MODY, Polygenic score, Autoantibody

## Abstract

**Aims:**

This study evaluates the contribution of common variants in Maturity-Onset Diabetes of the Young (MODY) genes on type 1 diabetes (T1D), using a polygenic score (PGS) approach.

**Methods:**

485 children and youth diagnosed with T1D from at least 1 year and 271 healthy controls (HC) were recruited. Personal information (i.e. age, sex, height, weight) were collected for each participant, and clinical information (i.e. age at diagnosis, disease duration, presence of autoantibodies and ketoacidosis at onset (DKA)) were also obtained for T1D subjects.

Participants were genotyped using Illumina Infinium Global Screening Array. PGS based on Single Nucleotide Polymorphisms (SNPs) in 16 MODY genes were developed. The association of this PGS with T1D susceptibility and clinical disease characteristics was assessed by regression analysis.

**Results:**

A PGS including 335 SNPs in MODY genes discriminates T1D from HC (AUC = 60.1%, AIC = 787.6). This PGS was significantly higher in T1D compared to HC (p-value = 0.0004, pseudo-R2 = 2.85%). Moreover, regression analysis between PGS and T1D clinical characteristics showed higher PGS values in T1D subjects with zinc transporter 8 autoantibodies (ZnT8A) compared with T1D subjects without ZnT8A (p-value = 0.04). A similar trend was also observed for antibodies directed against glutamic acid decarboxylase (GADA), although the association did not reach statistical significance (p-value = 0.06).

**Conclusions:**

Our study suggests that a polygenic approach based on MODY genes may discriminate T1D from HC and may contribute to patient stratification, helping to better understand T1D heterogeneity.

**Supplementary Information:**

The online version contains supplementary material available at 10.1007/s00592-025-02544-w.

## Introduction

Diabetes is considered a complex disorder with heterogeneous etiology, pathogenesis, clinical presentation, and outcomes.

T1D is the most common form of diabetes in children and youth [1]. T1D is a multifactorial disease characterized by autoimmune destruction of pancreatic beta cells in genetically predisposed individuals. The major susceptibility locus maps to the *HLA* class II genes at 6p21, although other susceptibility loci contributing to disease risk have been identified [2].

Another form of diabetes is MODY, often misclassified as T1D due to the young age at presentation. MODY is the most common type of monogenic diabetes, with autosomal dominant inheritance and caused by pathogenic variants resulting in pancreatic beta cell dysfunction, such as *HNF4A, GCK, HNF1A, PDX1, HNF1B, NEUROD1, KLF11, CEL, PAX4, INS, BLK, ABCC8, KCNJ11, APPL1*. The different MODY subtypes vary based on age at onset, treatment, and the presence of extra-pancreatic manifestations [3].

Although MODY and multifactorial forms of diabetes are distinct conditions, genes responsible for MODY were reported to be involved also in the development of type 2 diabetes (T2D) and gestational diabetes (GD) (4). Large-scale exome sequencing studies suggested, for example, an association between T2D risk and the aggregation of rare deleterious variants in MODY genes [5, 6], such as *GCK* gene. *GCK* causes MODY2 and common variants in this gene were also previously associated with increased risk of both T2D and GD [7–9]. Previous works have also assessed the role of variation in the *HNF1A* gene (responsible for MODY3) in susceptibility to both T2D and GD, suggesting that HNF1A may participate in pathways involved in abnormal blood glucose levels and that HNF1A variants may lack sufficient penetrance to cause diabetes but may still increase the susceptibility to T2D and GD [4–10]. Studies have also reported additional evidence for a relationship between T2D and other MODY genes, such as *KCNJ11* [11], *HNF4A* [12], and *ABCC8* [13].

In contrast to T2D, few works have been conducted to study the influence of MODY genes on T1D. For example, the *PAX4* gene, responsible for MODY9 and playing a crucial role in pancreatic beta cell development, is considered a T1D susceptibility factor [14, 15]. Although controversial results have emerged, an influence of *NEUROD1* (responsible for MODY6) on T1D has been also reported [16, 17]. Moreover, other studies suggested an influence of variants in the *KCNJ11* gene (associated with MODY13) on both T1D susceptibility and its clinical features (body mass index (BMI) at onset, insulin requirement, and C-peptide at onset) [18, 19]. In our previous work on the *HNF1A* gene, although we did not find an association between T1D, we found that an *HNF1A* SNP is associated with clinical characteristics of T1D, such as Hemoglobin A1C (HbA1c) levels and Insulin-dose adjusted glycated hemoglobin A1c (IDAA1c) [20].

Furthermore, SNPs in the *INS* and *INSR* genes, coding for preproinsulin precursor of insulin and insulin receptor respectively, were associated with T1D, as well as suboptimal glycemic control and the absence of anti-insulin antibodies in T1D [21].

Overall, this evidence suggests the existence of a genetic overlap between MODY and common multifactorial forms of diabetes. However, more studies with larger sample sizes are needed to better understand the role of these genes on T1D. Moreover, a lack of comprehensive investigation on the polygenic effect of MODY genes in T1D remains. Therefore, in this study, we analyzed in a cohort of children and young adults the association of a PGS restricted to MODY genes with T1D susceptibility and clinical disease characteristics.

## Materials and methods

### Subjects

485 T1D individuals and 271 HC were recruited. Inclusion criteria for the T1D cohort were diagnosis of T1D from at least 1 year, age between 6 and 21 years, and absence of other types of diabetes (i.e., type 2, monogenic diabetes, cystic fibrosis-related diabetes). The exclusion criteria for HC were diagnosis of T1D or other diabetes forms, obesity and other metabolic disorders, HbA1c > 6% (> 42 mmol/mol), family history of diabetes.

HC and T1D individuals were enrolled at Emergency Departments and Diabetes Units of IRCCS Burlo Garofolo (Trieste, Italy), Regina Margherita Children’s Hospital (Torino, Italy), University Medical Center (Ljubljana, Slovenia), IRCCS San Raffaele (Milano, Italy), Santa Chiara Hospital (Trento, Italy), Pediatric Diabetology Center G. Stoppoloni (Napoli, Italy) and Maggiore della Carità Hospital (Novara, Italy).

The ethics committee approved the protocol (CEUR-2022-Em-175, KME-0120–65/2019/4). The study was conducted according to the Declaration of Helsinki and Good Clinical Practice. All participants and their parents (for participants aged < 18 years) gave written informed consent before enrollment.

### Demographic and clinical characteristics

Demographic and clinical information such as age, sex, height, and weight were collected for each participant. Standard deviation scores of body mass index (BMI SDS) were calculated according to WHO reference charts using the Growth Calculator 4 software (http://www.weboriented.it/gh4/).

For each participant, HbA1c was measured with finger pricks using portable instrumentation at outpatient clinics.

Moreover, for T1D participants, medical history (i.e. age at diagnosis, disease duration, presence of DKA at onset) and clinical information (i.e. insulin requirement) were obtained from medical records. For a sub-sample of T1D participants, the presence at the onset of the following antibodies was also available: Insulin-directed antibodies (IAA), antibodies directed against tyrosine phosphatase (IA-2A), GADA and ZnT8A.

### Genotyping and imputation

For each participant, a DNA sample was extracted from saliva by using the EZ1 DNA investigator kit (Qiagen, Milan, Italy).

Genotyping was conducted by using Illumina Infinium Global Screening Array (GSA v3.0), Illumina iScan instrument, and Illumina GenomeStudio software (V2.0, Illumina, Inc., San Diego, CA, USA). Quality control (QC) of genotyping data was performed to remove: (1) samples with call rate < 95%, sex discrepancy, heterozygosity outside 6 standard deviations (sd) from the mean, identity by descent (IBD) proportion > 0.4; (2) duplicate SNPs, SNPs with missing call rate > 1% or with Hardy–Weinberg equilibrium (HWE) p-value < 1 × 10^−6^ [22].

Genotype imputation was also performed using the software Eagle2 for genotype phasing [23], impute5 software [24] and IGRP panel [25] for missing genotype prediction after phasing. Variants with quality scores < 0.8 were removed, to take into account only well-imputed variants.

### PGS creation

In the present study, 688 samples and about 13.000.000 SNPs were available after QC and imputation. However, for PGS creation only SNPs within 16 genes previously associated with MODY, including syndromic diabetes genes (*HNF4A, GCK, HNF1A, PDX1, HNF1B, NEUROD1, KLF11, CEL, PAX4, INS, BLK, ABCC8, KCNJ11, APPL1, WFS1*, *INSR*), were selected (Table 1) [26].

**Table 1:** Genes related to monogenic diabetes

GENE NAME	GENE LOCUS	OMIM*	TYPE
HNF4Α	20q13.12	125,850	MODY 1
GCK	7p13	125,851	MODY 2
HNF1Α	12q24.31	600,496	MODY 3
PDX1	13q12.2	606,392	MODY 4
HNF1Β	17q12	137,920	MODY 5
NEUROD1	2q31.3	606,394	MODY 6
KLF11	2p25.1	610,508	MODY 7
CEL	9q34.13	609,812	MODY 8
PAX4	7q32.1	612,225	MODY 9
INS	11p15.5	613,370	MODY 10
BLK	8p23.1	613,375	MODY 11
ABCC8	11p15.1	610,374	MODY 12
KCNJ11	11p15.1	616,329	MODY 13
APPL1	3p14.3	616,511	MODY 14
WFS1	4p16.1	222,300	Syndromic diabetes
INSR	19p13.2	610,549	Syndromic diabetes

Only SNPs in 16 genes previously associated with MODY were considered for PGS computation.

*OMIM (Online Mendelian Inheritance in Man), an updated catalog of human genes and genetic disorders and traits (https://omim.org)

**Fig. 1:**
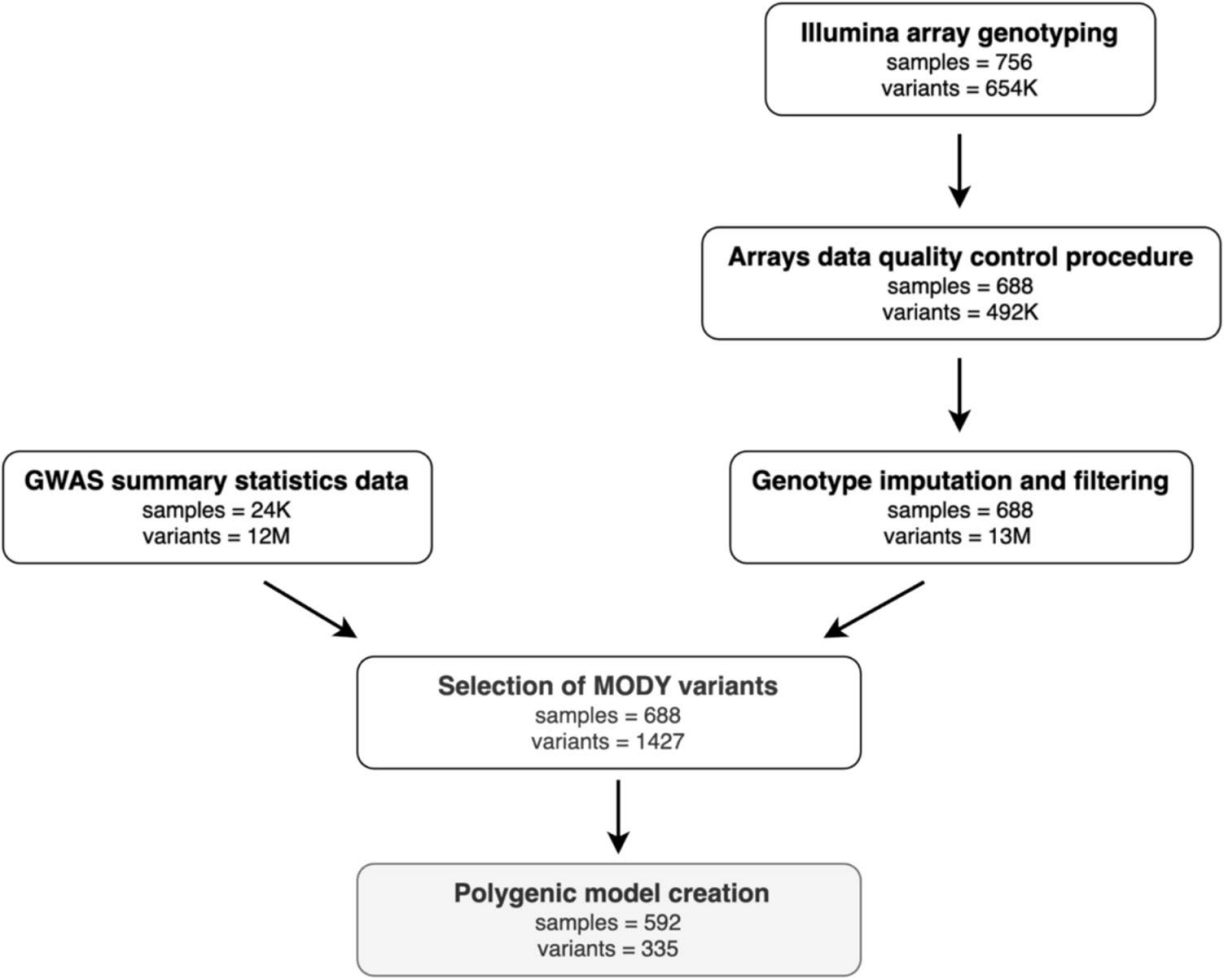
Workflow and data dimensionality at the end of any step**.** “Samples” indicates the numbers of participants, while “variants” indicates the numbers of SNPs. Matt grey rectangles report the final numbers of samples (n = 592) and variants included (n = 335) in the polygenic score analyses

Moreover, only common SNPs with minor allele frequencies equal to or greater than 0.01 were considered. Therefore, after this step, the number of individuals and variants was reduced from a total of 688 samples and 1427 SNPs to 592 samples and 335 SNPs, that were then used to calculate PGS. A detailed representation of the number of individuals and variants in any step was reported in Fig. 1.

For PGS creation the following steps were performed:

1. discovering step (i.e., finding and weighting of T1D associated markers) by using publicly available Genome Wide Association Study (GWAS) summary statistics, where only shared variants between GWAS summary statistics and our sample’s genotyping data were considered. In particular, we selected summary statistics of a T1D-GWAS performed on 24.840 individuals of European ancestry and included information on 9.061.522 SNPs (hg19 assembly) [27].
2. validation step (i.e., polygenic scoring computation) on our sample’s genotyping data, where the polygenic scoring computation was limited to ancestry-matched samples between T1D-GWAS and our data (i.e., European ancestry).

For scores creation, we tested different base and advance methods implemented inside the following software or R/Python packages: PLINK v1.9 [28], PRSice-2 [29], LDpred2 [30], lassosum [31], GCTA [32] and PRS-CS [33]. In base methods (such as PLINK or PRSice-2) only a set of independent SNPs and above a selected GWAS *p*-value threshold are utilized to calculate the score. While, advance methods (such as LDpred2 or PRS-CS) incorporate genome-wide SNPs and linkage disequilibrium (LD) information.

The different PGS models obtained were then compared for the best discrimination between case and control subjects. The best PGS model was selected by using the Akaike information criterion (AIC) value (comparing different models and determine which one is the best fit for our data) and area under the curve (AUC) metric obtained by ROC (receiver operating characteristic) curve analysis (i.e., the probability of a PGS from a random case being larger than a PGS from a random control).

### Statistical analyses

Descriptive statistics represent percentages, means, standard deviations (sd), median and interquartile range (IQR). To test the normality skewness and kurtosis were calculated. Sample characteristics between T1D and HC were assessed by Welch corrected t-test, Mann–Whitney test, and chi-square test, as appropriate.

The association of PGS (as independent variable) with T1D and clinical characteristics (such as HbA1c, DKA, age at onset) as outcomes was assessed by linear or logistic regression analyses, sex and age-adjusted. For some clinical characteristics (i.e. HbA1c and antibodies presence), disease duration was also included as a covariate in regression analyses.

For logistic regression analyses, the proportion of variance explained by PGS was inferred by using Nagelkerke pseudo-R2. Specifically, PGS pseudo-R2 adjusted for sex and age were obtained by subtracting the Nagelkerke pseudo-R2 value of the reference null model (i.e. the logistic regression model with only sex and age as predictors) from the Nagelkerke pseudo-R2 value of the complete model (i.e. the logistic regression model with PGS, sex and age as predictors).

Missing values in our available data were treated by using the pairwise deletion method instead of the listwise ones to reduce any statistical estimation bias related to the listwise deletion approach [34].

All statistical analyses were performed with R software (V4.2.2., www.r-project.org, accessed on 31 October 2022).

## Results

In this study, 389 participants with T1D and 203 HC, for in total 592 subjects (316 females and 276 males), were used for the PGS calculation.

No sex differences emerged among T1D and HC (p-value = 0.34): females were 52% among T1D and 56% among HC. On the contrary, age and BMI SDS differences were found: mean age was 13.9 ± 3.5 in T1D and 12.8 ± 3.9 in HC (p-value = 0.001); mean BMI SDS was 0.2 ± 1.0 in T1D and -0.4 ± 1.2 in HC (p-value < 0.001). Detailed overview of participants characteristics including T1D clinical features is reported in Table 2.

**Table 2:** Demographic and clinical characteristics in T1D and HC individuals

	HC	T1D	p-value
Sex (% females)	56%	52%	0.34
Age (years) (mean ± SD)	12.8 ± 3.9	13.9 ± 3.5	0.001
BMI SDS (mean ± SD)	− 0.4 ± 1.2	0.2 ± 1.0	< 0.001
HbA1c at enrollment %(mmol/mol) (median [IQR])	5.4 (36) [0.4]	7.5 (58) [1.4]	< 0.001
Age at diagnosis (years) (mean ± SD)	–	7.7 ± 3.9	–
Disease duration (years) (mean ± SD)	–	6.2 ± 3.9	–
Presence of DKA (% Yes)	–	39%	–
Insulin requirement (U/kg/day) (median IQR]))	–	0.8 [0.3]	–
IAA (% Yes)	–	60%	–
ZnT8A (% Yes)	–	30%	–
IA-2A (% Yes)	–	40%	–
GADA (% Yes)	–	79%	–

Differences were computed by Welch corrected t-test, Mann Whitney test, chi-square test, as appropriate

Standard deviation scores of body mass index (BMI SDS); Hemoglobin A1C (HbA1c); Ketoacidosis at onset (DKA); Insulin-directed antibodies (IAA); antibodies directed against tyrosine phosphatase (IA-2A); antibodies directed against glutamic acid decarboxylase (GADA); zinc transporter 8 autoantibodies (ZnT8A)

**Fig. 2:**
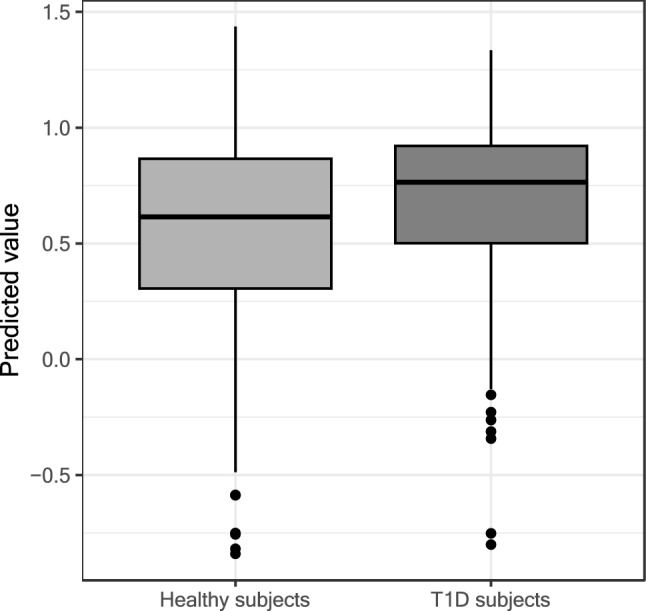
Boxplot of PGS in healthy subjects and T1D subjects. Y axed values represents the predicted values obtained by the logistic model used to infer the age and sex adjusted relation between T1D risk and the selected PGS. Type 1 Diabetes (T1D)

Among generated PGSs, the PGS that best discriminates T1D from HC was generated by PRS-CS method with a phi value of 0.0001 (AUC = 60.1%, AIC = 787.6). Age and sex-adjusted regression analysis showed that this PGS was significantly higher in T1D compared to HC (Fig. 2), with a p-value of 0.0004 and pseudo-R2 of 2.85%. The pseudo-R2 of the null model (i.e. the model with only sex and age information) used as a comparison was 1.45%, showing that including PGS in the regression analysis improved the model and better discriminated T1D from HC.

In supplementary materials the comparation of the different PGS models (Table S1) and the effect size of each of 335 SNPs included in PRS-CS (Table S2) were reported.

Boxplots display data distribution; the box covers the interquartile range, the line marks the median, and whiskers indicate variability. Outliers appear as dots.

**Fig. 3:**
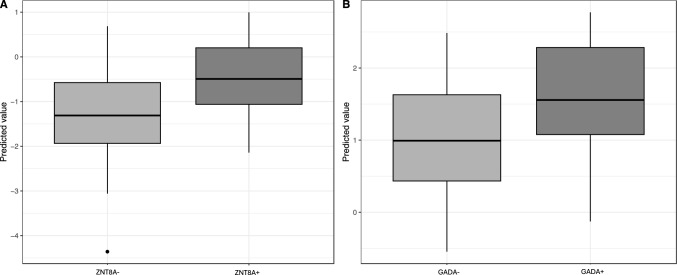
Boxplots of PGS in T1D subjects with and without ZnT8A (**A**) and GADA (**B**) autoantibody. Y axed values represent the predicted values obtained by the logistic model used to infer the age and sex adjusted relation between autoantibody and the selected PGS. Positive antibodies directed against glutamic acid decarboxylase (GADA +); negative antibodies directed against glutamic acid decarboxylase (GADA-); positive zinc transporter 8 autoantibodies (ZnT8A +); negative zinc transporter 8 autoantibodies (ZnT8A-)

Age and sex-adjusted regression analysis between PGS and T1D clinical characteristics were also performed. We found higher PGS values in T1D subjects with ZnT8A compared with T1D subjects without ZnT8A (p-value = 0.04), as shown in Fig. 3A. A similar trend was also observed for GADA autoantibody, although the association did not reach statistical significance (p-value = 0.06) (Fig. 3B).

No associations were found from the analysis of other clinical features of T1D subjects. Results are reported in Table S3.

Boxplots display data distribution; the box covers the interquartile range, the line marks the median, and whiskers indicate variability. Outliers appear as dots.

## Discussion

In the present study, we investigated the association of a PGS based on variants within 16 MODY genes with T1D and its clinical characteristics. We showed that PGS values are significantly higher in T1D individuals compared to healthy controls, suggesting that a PGS restricted to MODY genes may help to discriminate individuals with type 1 diabetes risk. Therefore, our results support the existence of shared genetic characteristics between monogenic and multifactorial forms of diabetes and confirm that a set of genes implicated in Mendelian forms can also be relevant in the pathogenesis of more common diseases, including T1D [35]. Diabetes is, in fact, a typical example of a disease in which multifactorial and monogenic forms may be considered extreme points of a spectrum of phenotypes caused by alleles ranging from strong to weak effects, from low to high frequency, and from coding to regulatory sites [36]. However, while in T2D there is extensive evidence, few studies with sometimes contradictory results are published on the relationship between T1D and MODY genes. Moreover, these studies have only assessed the contribution of one or a few SNPs in MODY genes. Our study is likely the first that used a polygenic approach for developing a PGS restricted to MODY variants and analyzed its contribution on T1D risk and clinical characteristics.

The PGS combines several SNPs into a single aggregated score, based on the number of risk variants that a person carries, weighted by SNP effect sizes that are derived from an independent large-scale discovery GWAS [37]. Several whole-genome PGS for T1D, including both *HLA* and no *HLA* variants, have been already developed in previous studies [38, 39], highlighting that the development of PGS may be effective in the discrimination between T1D and other forms of diabetes, but also in improving risk stratification of presymptomatic T1D [40]. Our result provides additional evidence, reporting that also a PGS based on MODY genes may allow to discriminate T1D risk. In particular, we observed that the variance explained (in terms of R2) by the regression model including sex, age and PGS is higher in respect to a model without PGS, in accordance with data previously reported in a study using another set of genetic variants [41]. Therefore, given the cost-effectiveness of SNP genotyping, our findings suggest that the integration of PGS on variants in selected MODY genes could have clinical and research potential in T1D and could be used as an additional source of information in pre-T1D.

In the present work, we also analyzed the possible association of the PGS bases on MODY genes and clinical characteristics, such as HbA1c, DKA, and disease duration. In a sub-sample of T1D participants for which information on the presence of diabetes-related autoantibodies was available, we found higher values of our selected PGS in T1D subjects with ZnT8A compared with T1D subjects without ZnT8A. A similar trend was also observed for GADA.

This result suggests that PGS, in addition to discriminating T1D from healthy controls, may also be important for gaining a better understanding of disease heterogeneity (in terms of the presence of autoantibodies) and, eventually, for the assignment of personalized disease management.

T1D is characterized by the destruction of insulin-producing beta cells in pancreatic islets due to an autoimmune attack, that is mirrored by the appearance of specific autoantibodies. Genetic factors are among the determinants that may influence the presence of autoantibodies in T1D. For instance, the association of certain *HLA* haplotypes with the occurrence of islet autoantibodies is consistent in various studies [42, 43]. Moreover, other studies have reported a contribute of other genetic factors in development of islet autoantibodies, including MODY genes, such as *INS,* that is considered the second strongest risk locus after HLA for T1D. In past studies polymorphisms in the *INS* gene were associated with decreased insulin expression in the pancreas and especially in the thymus, where self-antigens are processed, influencing the selection of T cells that play a critical role in beta cell autoimmunity [44]. While, a very recent study have placed beta cells at the heart of the disease process leading to T1D, suggesting that *INS* polymorphisms may act instead of or in concert with the previously proposed mechanism. In particular, authors showed that a protective *INS* SNP (rs3842752) causes accelerated inositol-requiring enzyme 1α (IRE1α)-dependent decay of insulin mRNA, reducing ER stress and beta cell immunogenicity [45].

To our knowledge, studies focused on ZnT8A or GADA in T1D have not been conducted. Therefore, we cannot propose any explanation for the association between PGS from MODY genes and the presence of ZnT8A revealed in the present work. However, we can speculate that variants on MODY genes may contribute to the initiation of beta cell autoimmunity or the progression of beta cell destruction since many of the genes included in the PGS are involved in these mechanisms.

Future works should be carried out to confirm the observed trend in a larger sample size and to better elucidate genetic mechanisms that play a role in the development of autoimmunity in T1D.

The present study had certain limitations. First, some clinical information was not available for all participants, limiting, for example, the analysis of the association of PGS with autoantibodies in 149 T1D subjects. Second, only common SNPs were included in the PGS, because of the moderately low number of participants for a genetic study. Therefore, further research using large datasets are required to confirm our results.

Despite these limitations, our study is the first to use a polygenic approach to study the joint contribution of MODY genes on T1D and its clinical characteristics, suggesting that a PGS restricted to MODY genes may contribute to the identification of individuals at high genetic risk of T1D and may help to better understand T1D heterogeneity.

## Supplementary Information

Below is the link to the electronic supplementary material.

Supplementary file1 (DOCX 18 KB)

Supplementary file2 (XLS 67 KB)

## Data Availability

Some or all data generated during and/or analyzed during the current study are not publicly available but are available from the corresponding author upon reasonable request.
