# Supplementary material for: Polygenic score from MODY genes is associated with type 1 diabetes and disease characteristics
Source: Acta Diabetol. 2025 Jun 20;62(11):2021–9. doi: 10.1007/s00592-025-02544-w (PMC12640318; doi:10.1007/s00592-025-02544-w)
Supplement: Supplementary file 1 — Supplementary file1 (DOCX 18 KB) [file 592_2025_2544_MOESM1_ESM.docx]

| **PGS Model** | **AIC** | **AUC** | **SNPs number ^a^** |
| --- | --- | --- | --- |
| PLINK | 796.761 | 57.887 | 90 |
| PRSice-2 | NA | NA | NA |
| Lassosum | 786.053 | 60.626 | 28 |
| PRS-CS | 787.613 | 60.141 | 335 |
| LDpred2 | NA | NA | NA |
| GCTA | NA | NA | NA |

**Table S1. Comparation among tested PGS models.**

Polygenic score (PGS); Akaike information criterion (AIC); Area under the receiver operator curve (AUC)

^a^ SNPs number utilized by the software to calculate the score. Base methods (such as PLINK or PRSice-2) includes only a set of independent SNPs above a selected GWAS *p*-value threshold, while advance methods (such as LDpred2 or PRS-CS) incorporate genome-wide SNPs.

NA is reported for models including a very low number of SNPs or that can’t be computed by the software.

|  | **BMI-SDS** | **HbA1c at enrollment** | **Age at diagnosis** | **Presence of DKA** | **Insulin requirement** | **IAA** | **ZnT8A** | **IA-2A** | **GADA** |
| --- | --- | --- | --- | --- | --- | --- | --- | --- | --- |
| PGS |  | 0.54 | 0.99 | 0.15 | 0.66 | 0.80 | **0.04** | 0.43 | 0.06 |
| Age |  | 0.90 | 0.65 | 0.43 | 0.75 | 0.66 | 0.44 | 0.68 | 0.79 |
| Sex |  | 0.39 | 0.50 | 0.28 | 0.56 | 0.43 | 0.35 | 0.40 | 0.66 |
| Disease duration |  | 0.44 | - | - | 0.74 | 0.63 | 0.34 | 0.54 | 0.57 |

**Table S3. P-value from association analysis between PGS and clinical characteristics among T1D subjects.**

Disease duration was used as covariates only for some clinical characteristics.

Clinical characteristics are available for all T1D collected (n=485), expect for autoantibody presence (n=149).

Polygenic score (PGS); Standard deviation scores of body mass index (BMI SDS); Hemoglobin A1C (HbA1c); Ketoacidosis at onset (DKA); Insulin-directed antibodies (IAA); antibodies directed against tyrosine phosphatase (IA-2A); antibodies directed against the cytoplasm of pancreatic islet cells (ICA); antibodies directed against glutamic acid decarboxylase (GADA); zinc transporter 8 autoantibodies (ZnT8A).
